# Differences in Management of Neonates with Hypoxic–Ischemic Encephalopathy (HIE) by Level of Neonatal Care Provided at Birth: Insights from a Referral-Based Cohort in the Canton of Zurich, Switzerland

**DOI:** 10.3390/children13010142

**Published:** 2026-01-19

**Authors:** Ladina Erni, Ariane Pfister, Christian Haslinger, Michael Kleber, Barbara Brotschi, Dirk Bassler, Vinzenz Boos, Beate Grass

**Affiliations:** 1 Newborn Research, Department of Neonatology, University Hospital Zurich, University of Zurich, 8091 Zurich, Switzerland; 2 Department of Neonatology, Hospital Zollikerberg, 8125 Zollikerberg, Switzerland; 3 Department of Obstetrics, University Hospital Zurich, University of Zurich, 8091 Zurich, Switzerland; 4 Center for Pediatric and Adolescent Medicine, Neonatology, Cantonal Hospital Winterthur, 8400 Winterthur, Switzerland; 5 Department of Intensive Care and Neonatology, University Children’s Hospital Zurich, 8008 Zurich, Switzerland; 6 Children’s Research Center, University Children’s Hospital of Zurich, University of Zurich, 8008 Zurich, Switzerland

**Keywords:** hypoxic–ischemic encephalopathy, delivery facilities, level of neonatal care, neonatal transport, healthcare organization and infrastructure

## Abstract

**Background/Objectives**: Neonates with hypoxic–ischemic encephalopathy (HIE) are born in delivery facilities with different levels of neonatal care. The objective of this study was to investigate differences in the incidence of HIE and postnatal management between different levels of neonatal care in delivery facilities. **Methods**: This is a retrospective, multi-center cohort study of neonates with moderate-to-severe HIE receiving therapeutic hypothermia (TH) in the Canton of Zurich, Switzerland, registered in the Swiss National Asphyxia and Cooling Register between 2015 and 2023. Incidences of HIE receiving TH were calculated for all delivery facilities according to the national levels of neonatal care on site (Level I—basic; Level IIB—intermediate (no Level IIA facility in the Canton of Zurich); Level III—intensive neonatal care). Perinatal characteristics and variables on transport and outcomes were compared between neonates born in Level I and Level IIB facilities (the majority of the HIE population) and reported for neonates born in all other facilities (for completeness). **Results**: A total of 173 neonates (79 (45.7%) born in Level I; 80 (46.2%) in Level IIB; 9 (5.2%) in Level III; 5 (2.9%) in birthing centers) were admitted to a neonatal cooling center to receive TH. The average number of annual cases of HIE receiving TH per facility was 0.67 (0.11–1.50) in Level I and 2.22 (0.22–3.11) in Level IIB facilities (*p* = 0.088), respectively. There was no difference in Apgar score, worst pH (within 60 min after birth) and the severity of encephalopathy between neonates born in Level I and Level IIB facilities. Neonatal transport team requests were initiated earlier in Level I facilities (median 12 vs. 34 min of life, *p* < 0.001). There was no difference in age at initiation of TH (median 3 vs. 3 h, *p* = 0.431) and the time when target temperature was reached (median 4 vs. 4 h, *p* = 0.431) between neonates born in Level I and Level IIB facilities. **Conclusions**: The level of neonatal care available in delivery facilities influenced the management of neonates with HIE receiving TH.

## 1. Introduction

### 1.1. Background

Hypoxic–ischemic encephalopathy (HIE) is associated with insufficient oxygen supply and/or reduced perfusion of the most vulnerable organs—especially the brain—in the peri-, intra- and postpartum period. To date, therapeutic hypothermia (TH) is the only evidence-based neuroprotective treatment in (near-)term neonates with moderate and severe HIE with a reduction in mortality and improvement in neurodevelopmental outcomes at 18–24 months of age [1,2,3]. HIE has an incidence of 1 to 8 per 1000 live births in highly industrialized countries and often occurs unexpectedly in term or near-term neonates in previously unremarkable pregnancies with often no specified risk factors [4]. Different levels of neonatal care are available at delivery facilities, and thus, neonates often require postnatal transport to neonatal cooling centers to be evaluated for TH [5].

In Switzerland, levels of neonatal care are categorized according to national criteria by the Swiss Society of Neonatology [6] (Level I basic, Level II A/B intermediate, Level III 58 intensive care). Moreover, as of 2011, all term and near-term neonates (gestational age ≥ 35 0/7 weeks) with HIE are registered in the Swiss National Asphyxia and Cooling Register [7]. TH is offered for moderate-to-severe HIE in neonatal cooling centers, which in most cases, mandate postnatal transport by neonatal transport teams, since the majority of neonates with HIE are outborn [5]. Previous studies in different healthcare settings reported that the place of birth—i.e., inborn versus outborn and the level of available neonatal care on site—was of significance regarding the extent of brain injury and neonatal outcomes in HIE receiving TH [5,8].

### 1.2. Rationale

We therefore aimed to investigate the incidence of HIE and postnatal management according to the national levels of neonatal care in the Canton of Zurich, Switzerland. The primary goal was to depict local healthcare organizational factors of obstetric and neonatal care provision, including details on transports to the neonatal cooling centers, for neonates born in all types of delivery facilities, including birthing centers and hospitals with all levels of neonatal care.

## 2. Materials and Methods

### 2.1. Study Design, Setting, and Participants

This was a retrospective, multi-center referral-based cohort study of prospectively collected data of neonates with moderate-to-severe HIE receiving TH in the Canton of Zurich, Switzerland, registered in the Swiss National Asphyxia and Cooling Register between 2015 and 2023. Neonates with mild HIE were not included.

TH was provided in three neonatal cooling centers in the Canton of Zurich: University Hospital Zurich (Level III neonatal intensive care unit (NICU); inborns and outborns), University Children’s Hospital Zurich (Level III neonatal care in pediatric intensive care unit; outborns only), and Cantonal Hospital Winterthur (Level IIB neonatal intermediate care; inborns and outborns) [6]. In addition, there were 14 delivery facilities providing Level I neonatal care (basic neonatal care of the healthy, (near-)term neonate in a mother–baby unit; no pediatrician on site), 3 hospitals with Level IIB neonatal care (intermediate neonatal care, ≥32 weeks gestational age, non-invasive respiratory support; neonatologist on site) and 2 birthing centers (midwife care) [6]. All these institutions regularly referred neonates to one of the three neonatal cooling centers in the Canton of Zurich if indicated. There was no Level IIA neonatal care facility in the Canton of Zurich [6]. Attending a structured neonatal resuscitation training course (“Start4Neo”; [9]) every two years was mandatory for all professions with regular attention to deliveries. Neonatologists working in facilities with different levels of neonatal care and obstetricians (no national obstetric grading of delivery facilities in Switzerland) collaborated in this project [10].

### 2.2. Data Sources, Data Collection and Variables

To calculate the incidences of HIE, data on the annual number of liveborn neonates in each delivery facility between 2015 and 2023 was provided by the Department of Health of the Canton of Zurich, Switzerland [11]. For the delivery facilities located outside the Canton of Zurich with regular referrals to a neonatal cooling center within the Canton of Zurich (in the absence of a neonatal cooling center within the canton of origin), we collected the numbers of births from the annual reports of the hospitals [12,13,14].

All included neonates ≥ 35 weeks of gestation were registered in the Swiss National Asphyxia and Cooling Register with written parental informed consent, including the further scientific use of data. All neonates were treated with whole-body cooling initiated within 6 h of birth, targeting 33.0 °C to 34.0 °C core temperature, and continued for 72 h, according to the Swiss National Asphyxia and Cooling Register Protocol [7]. Inclusion and exclusion criteria for TH according to the national cooling protocol were applied [7,15]. All neonates had continuous (amplitude-integrated) electroencephalography monitoring during hypothermia and rewarming; however, the pattern of the (amplitude-integrated) electroencephalogram was not an inclusion criterion for TH according to the national cooling protocol. Register data was amended manually by an electronic neonatal chart review if general hospital consent was available in the three participating neonatal cooling centers. For feasibility reasons, i.e., the very limited number of HIE cases per delivery facility and the need for ethical approval in all referral hospitals, maternal obstetric charts were not available for review.

The following variables were included:

#### 2.2.1. Maternal Variables

Maternal age and maternal morbidities (obstetric, gynecologic, neurologic, psychiatric, infectious, endocrine, autoimmune); social background, medication and substance use (smoking, alcohol, cannabis, other drugs) and current and previous pregnancy demographics and outcomes (including fertility treatment and regular pregnancy check-ups). The mother’s medical history was considered unremarkable if there were no charted diagnoses or medications.

#### 2.2.2. Neonatal Variables

Birth place (level of neonatal care according to the Swiss Society of Neonatology [6]), birth details and neonatal demographics, details on neonatal resuscitation and criteria for HIE according to the national flowchart in the appendix (Appendix A) to initiate TH were included. The latter comprised the so-called “A-criteria” (APGAR ≤ 5 at 5 (10) min; need for ventilation at 10 min; worst pH, base deficit and lactate within the first 60 min of life) and “B-criteria” (severity of encephalopathy, assessed by Sarnat score [16] and/or Thompson score [17]; seizures). Passive cooling was started during neonatal transport after confirming the indication of TH by a transport physician (consultant neonatologist). Time to initiate TH and time to reach target temperature, neurologic prognostic factors during TH (evolution of Sarnat and/or Thompson score, seizures, persistently elevated lactate [18]) and mortality were reported. Brain injury on magnetic resonance imaging and pathology (neuropathology and placental pathology) were not evaluated in this study. Long-term neurodevelopmental outcomes were not included.

#### 2.2.3. Healthcare Organizational Factors and Infrastructure of Delivery Facilities

The available level of neonatal care on site; weekday and time of delivery; medical professionals present during birth (midwife, obstetrician, anesthesiologist, pediatrician in domo) and their clinical experience (board certification in subspecialty, title, clinical position); time at notification of the neonatal transport team; arrival time of the neonatal transport team on site; and travel distances between referral site and neonatal cooling center were measured.

### 2.3. Statistical Methods, Ethics and Reporting

All missing patient information was reported. All statistical analyses were conducted using R v4.4.1 (the R Foundation for Statistical Computing, Vienna, Austria). Descriptive data were reported as frequency (proportion) for categorical variables and as median (interquartile range) or mean (range) for continuous variables. Groups were compared using Pearson’s chi-squared and Fisher’s exact test for dichotomous variables and the Wilcoxon rank-sum test for continuous variables in unadjusted analysis. A *p*-value of <0.05 was considered statistically significant.

Data collection, analysis and publication was waived by the Swiss ethical committee of the Canton of Zurich (KEK-ZH number 2024-01053) as it was considered a quality improvement project. STROBE guidelines were followed for reporting this observational study.

## 3. Results

### 3.1. Participants and Descriptive Data on Incidence of HIE Receiving TH

**Figure 1:**
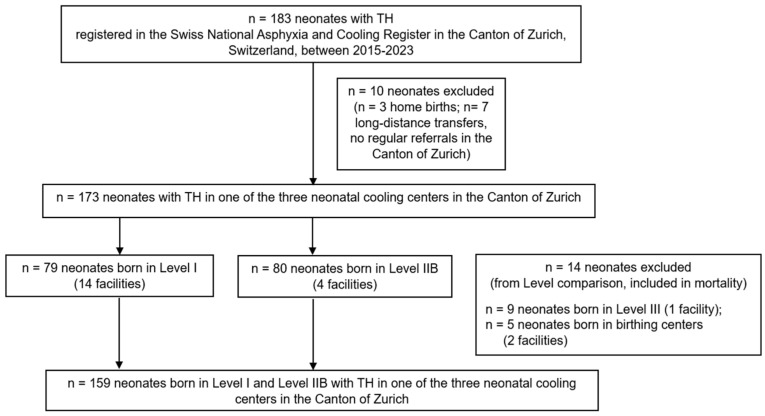
Flowchart of study population.

In total, 183 neonates with TH were registered in the Swiss National Asphyxia and Cooling Register between 2015 and 2023 in the Canton of Zurich, Switzerland. Ten neonates were excluded from this study: three midwife-attended home births and seven neonates who were born in facilities without routine referrals to the cooling centers in the Canton of Zurich. The remaining 173 neonates (173/183; 94.5%) were born in delivery facilities with regular referral to one of the three neonatal cooling centers in the Canton of Zurich, which provided TH to 50, 84 and 39 neonates in the study period, respectively (Figure 1).

Of those 173 neonates receiving TH, 79 (45.7%) neonates were born in one of 14 facilities with basic neonatal care (Level I) and 80 (46.2%) neonates were born in one of 4 facilities with intermediate neonatal care (Level IIB). Nine (5.2%) neonates were born in one facility with neonatal intensive care unit (Level III), and five (2.9%) neonates were born in two birthing centers (Figure 1). In addition to the very limited corresponding sites with a single Level III facility and two birthing centers, the numbers of cases with HIE were small in both settings (Supplemental Table S1), and thus, those neonates were excluded from further analysis (except for reporting of incidence) due to limited generalizability, resulting in a final study cohort of 159 neonates (Figure 1).

Among the Level I and Level IIB facilities, there was no significant difference in the rate of neonates with HIE receiving TH, with an incidence of 0.96 per 1000 births in Level I and 1.19 per 1000 births in Level IIB facilities (*p* = 0.195). The incidence of HIE receiving TH was 0.37 per 1000 births in the Level III facility (Table 1).

### 3.2. Outcome Data and Main Results

#### 3.2.1. Neonatal, Maternal and Delivery Characteristics

Among the 159 neonates, approximately half were delivered in Level I (49.7%) and Level IIB facilities (50.3%) (Table 2). They were evenly distributed in view of neonatal demographics (such as weight, gestational age, sex) and maternal characteristics (such as age, parity, co-morbidities, substance use). Social demographics did not differ between groups. Oligohydramnios was significantly more present in deliveries at Level IIB compared to Level I facilities (11.3% versus 0%, *p* = 0.003). Cardiotocography monitoring was used in almost all deliveries; however, initiation, duration and details on interpretation were not sufficiently available from the neonatal medical charts. The rate of perinatal sentinel events was similar in both groups. Delivery mode did not differ between the levels with a cesarean section rate of almost 50% in both groups (none due to polytrauma or maternal resuscitation).

Occurrence of HIE was evenly distributed between weekdays and weekends, with no evidence of circadian clustering (i.e., during off-hours). Delivery attendance of obstetric and anesthesia staff was comparable between groups. As expected, according to CANU levels of neonatal care [6], pediatric staff was more frequently present at birth in Level IIB compared to Level I (86.3% versus 5.1%; *p* < 0.001).

There were no significant differences between neonates born in Level I compared to Level IIB facilities regarding the so-called “A-criteria”, namely in the APGAR at 5 (10) min, worst pH/umbilical artery pH, base deficit and lactate within the first 60 min. However, more neonates received neonatal resuscitation (defined as any form of ventilation necessary at the age of 10 min) in Level I compared to Level IIB facilities (64.6% versus 44.9%; *p* = 0.020) (Table 3).

For the definitions of maternal variables, we considered the mother not to be obese unless it was specifically mentioned, or the documented body mass index was 30 or more. We investigated the circumstances of the birth: delayed access to care, defined by anything other than onset of labor in a hospital; abnormal birth setting (i.e., outside a delivery facility); maternal COVID-19 infection during birth; maternal polytrauma leading to imminent delivery. We reported evidence of CTG monitoring during labor and a protracted second stage of labor (>120 min) binarily. We further documented the CTG in categories of “normal”, “suspect” and “pathologic”. The mode of delivery was categorized into “spontaneous vaginal birth—cephalic”, “spontaneous vaginal birth—breech”, “instrumental delivery”, “elective cesarean section”, “secondary caesarean section” and “emergency caesarean section”, and in the case of the last three, why the surgical intervention occurred and if the mother received general anesthesia with endotracheal intubation or not. Obstetric decision times were included if available (minutes between decision for section and birth, minutes between uterotomy and birth, complications during delivery, placental abnormalities) and if the mother refused the recommended treatment (with delayed intervention). We categorized the mother’s origin into German-speaking countries (Germany, Austria, Switzerland) and others and determined the mother’s ability to converse and understand medical information in a language generally spoken in Switzerland (German, English, French, Italian).

#### 3.2.2. Variables on Neonatal Transport from Delivery Facility to Neonatal Cooling Center

One of the three neonatal cooling centers was classified as neonatal Level IIB [6] and was qualified as a neonatal cooling center for both inborn (i.e., requiring no transport, n = 22) and outborn neonates. Therefore, only 137 of the 159 (86.2%) neonates born in Level I or other Level IIB facilities had to be transferred after birth to a neonatal cooling center. Among these neonates, Level I facilities called the neonatal transport team of the corresponding cooling center significantly earlier than Level IIB facilities (12 (7, 23) min versus 34 (23, 50) min; *p* < 0.001), resulting in an earlier arrival of the transport team on site (56 (44, 68) min versus 74 (58, 93) min of life; *p* < 0.001) despite a longer travel distance (by ground) to Level I facilities compared to Level IIB facilities (22 (15, 31) km versus 6 (6, 6) km; *p* < 0.001) (Table 4).

Importantly, TH was initiated at a median 3 h of life (*p* = 0.431), and the target temperature of TH was reached at a median 4 h of life (*p* = 0.547) in both groups (Table 4).

#### 3.2.3. Neurologic Prognostic Factors

There was no persistent difference regarding the severity of encephalopathy (“B-criteria” of HIE), assessed with Sarnat and/or Thompson scoring [16,17], as well as no difference in lactate peak values, before the initiation of TH and during the course of TH between neonates born at Level I and Level IIB facilities (Table 5). However, there was a trend to more seizures on day 2 of life in neonates born in Level I facilities (19.7% versus 7.7%, *p* = 0.052). There were no readmissions after initial discharge from the NICU. The overall mortality was 15.6% (27/173).

## 4. Discussion

### 4.1. Statement of Principal Findings

This retrospective, multi-center cohort study of neonates with moderate-to-severe HIE receiving TH in the Canton of Zurich, Switzerland, registered in the Swiss National Asphyxia and Cooling Register between 2015 and 2023, confirmed that moderate and severe HIE occurred anywhere and anytime. The overall incidence of HIE receiving TH per 1000 live births was comparable between Level I and Level IIB facilities.

Maternal, pregnancy and delivery demographics, as well as most neonatal characteristics, including the severity of HIE and timing of initiation of and reaching target temperature of TH, did not differ between the neonates born in Level I compared to Level IIB facilities. All outborn neonates born in Level I facilities and the majority of neonates born in Level IIB facilities needed to be transported to a neonatal cooling center for TH. Being outborn was known to be associated with death and unfavorable neurodevelopmental outcome in the HIE population compared to inborn neonates (i.e., being born with HIE and treated with TH in same the neonatal cooling center) [5,8].

### 4.2. Interpretation Within the Context of the Wider Literature

These findings can be attributed to two main categories: patient-related factors and healthcare organizational factors (human/system/infrastructure) [19].

Since maternal, pregnancy and delivery characteristics, as well as neonatal characteristics, were similar, patient-related factors per se appeared comparable. However, being aware of patient-related risk factors [20,21,22,23] can alter anticipation and situational awareness, although HIE is multifactorial in origin and its cause often undetermined and not preventable, as commonly more than one factor contributes to a poor outcome [23]. In this study, for instance, cardiotocogram readings were not rigorously reported, similar to comparable studies [8]. Despite the incidence of perinatal sentinel events being similar in both Level I and Level IIB facilities and comparable to the literature [24], the reaction times from perinatal sentinel events to delivery remained unclear. Therefore, there might be differences in human responses and discrepancies due to system-related factors. Rapid recognition of HIE in the neonates born in Level I and Level IIB facilities was reflected in the timely initiation of the transport requests. Appropriate immediate neonatal resuscitation remained key [25], indicating the need for 24/7 availability of skilled personnel for neonatal resuscitation, which might explain why the call for help by the neonatal transport team was initiated earlier in Level I facilities, where pediatric staff was usually not present at birth. Awareness of local settings was thus incorporated in the medical care process to ensure professional neonatal care.

Neonates with HIE receiving TH born in Level I facilities received more respiratory support (resuscitation was defined as any form of ventilation necessary at the age of 10 min) compared to those born in Level IIB facilities. There was no difference in the objective markers of neonatal illness compared to, for instance, lower pH measurements in Level I facilities in other studies [8].

In our cohort, the overall mortality rate of 15.6% was comparable with other reports, ranging from 15 to 25% [26]. Mortality in HIE was often in the context of redirection of care [26], i.e., reflecting more severe brain injury [27]. In addition to objective neurodevelopmental prognostic factors, parental counseling on neurodevelopmental outcomes and shared decision-making in the neonatal cooling center might also explain why mortality differed in different settings [26]. Mortality was thus likely multi-factorial, and (due to small numbers) cannot be attributed to the level of neonatal care.

### 4.3. Implications for Policy, Practice and Research

This project was a collaboration of obstetricians and neonatologists (working in facilities of different level of neonatal care) and highlighted the importance of the continuum of care in the clinical pathway in neonates with HIE. In the Canton of Zurich, the continuum of care consisted of regular and readily accessible neonatal resuscitation training (“Start4Neo”; [9]), which established the close collaborations of Level I facilities with Level IIB and Level III facilities as well as a clear allocation of the responsible neonatal transport team. The local neonatal transport infrastructure in the Canton of Zurich was very satisfactory, with all targets of TH being met [28], which strongly depended on local infrastructures and geography in other reports [8,29].

Nevertheless, adherence to evidence-based interventions for obstetric care and neonatal resuscitation guidelines remained unquestionable [9]. This study supported the implementation of local quality improvement measures for the care of neonates with HIE, integrating human and system factors in a local context to be effective.

The potential lack of sufficiently trained medical personnel in neonatal resuscitation due to organizational standards warranted further investigation. Resuscitation and simulation team training was reported to reduce mortality and seizures in neonates with HIE [30]. However, despite regular mandatory structured neonatal resuscitation training for all birth-attending professions in Switzerland (“Start4Neo”; [9]), attendance was recorded centrally but attendance was not followed up, and thus non-attendance was not sanctioned.

In Switzerland, there is no national obstetric grading of delivery facilities, in contrast to the assigned national levels of neonatal care [6]. In the Canton of Zurich, obstetric facilities are categorized in the so-called “hospital list” [10]. Early anticipation of maternal or obstetric risk factors is of importance; however, many birth-related complications might happen shortly before delivery and antenatal transfer in view of risk factors might often not be possible in HIE [8]. Due to the short travel distances in Switzerland, the standby provision of delivery for neonatal transport teams could be an option if the anticipated emergency situation could be identified beforehand (e.g., dispatching the transport team with the onset of the perinatal sentinel event). In recent years, telemedicine and online applications were moved forward to guide less experienced teams through neonatal resuscitation [27,31].

Our results and previously published reports depicted wide inter-facility variability in incidence of HIE despite the same level of neonatal care [8]. In this study, the lowest reported incidence of HIE occurred in a Level III facility; however, there were single facilities with comparable reported incidences in some Level I and Level IIB facilities. This suggested that human- and system-related factors were key. Further research is needed to understand the interplay of various perinatal factors and to identify those at the highest risk for HIE with the aim to intervene early. Despite different medical care mandates as per the level of neonatal care, the utmost goal should be to standardize care in neonatal resuscitation and the initial management of neonates with HIE receiving TH [8].

### 4.4. Strengths and Limitations

This study was the first to analyze the epidemiology of neonates with HIE receiving TH in the Swiss health setting with nationally defined levels of neonatal care [6]. The study did not address causality. It benefitted from the availability of and review of all births and all reported cases of HIE receiving TH in the Canton of Zurich, Switzerland. The results should be generalizable across Switzerland, and are presented according to the levels of neonatal care without deducing individual facilities.

All neonates were registered in the Swiss National Asphyxia and Cooling Register, which is known for standardized, complete and comprehensive data entry over the entire study period. For example, there was almost complete data on cord gases compared to other healthcare settings [29]. However, this cohort included neonates in ≥35 + 0 weeks of gestation” to provide clarity and precision, and we acknowledge that the Sarnat [16] and Thompson [17] classifications were validated for neonates in 36 and 37 weeks of gestation. By design and for feasibility reasons, pediatric neuroradiology, neuropathology, placental pathology, neurogenetics and developmental pediatricians were not used in collaboration in this study. This study was limited by neonatal chart review (until discharge from the NICU, not including neurodevelopmental follow-up data) and having no access to maternal medical charts. The documentation thus missed some maternal health data and obstetric characteristics, such as detailed information on cardiotocograms and decision-to-delivery times. According to the literature, the definition of emergency cesarean section might vary, and there could have been systemic differences between facilities with different levels of neonatal care [8].

There might be underlying population-based disparities in antenatal and postnatal referral patterns, e.g., based on the place of residence reflected in patient referrals to different delivery facilities, and also regarding the varying mandates of medical care provision (such as case mix index) at the different facilities. For instance, facilities with Level III neonatal care might focus on extremely premature infants and neonates with a predicted need for postnatal support, whereas facilities with Level I neonatal care primarily care for (near)-term deliveries with no anticipated maternal or neonatal complications, as often occurs in HIE.

In addition, some cases of HIE may have been neonates who were born at a certain facility, but whose mothers were transferred only shortly before giving birth. It is therefore not possible to make any statements about the quality of obstetric care in this retrospective study. Surprisingly, documentation on perinatal surveillance, staff present at delivery and their involvement in neonatal resuscitation was overall not stated in full detail, and would have been very valuable to distinguish a lack of team skills, communication or local resources. The perinatal care and perinatal surveillance was inadequately documented; however, we cannot determine if it was conducted according to present-day standards or not. The incomplete and deliberate documentation of the neonatal resuscitation team members and assigned tasks appeared to be caused by the highly stressful resuscitation and referral situation. From a medico-legal point of view, however, it is difficult to understand, and in fact unacceptable, why this central aspect of documentation is often disregarded by obstetric and neonatal teams. Further research, using semi-structured interviews, was already initiated to investigate level-based infrastructures and resources for neonatal resuscitation in more detail.

This study was designed to investigate the management of neonates with moderate-to-severe HIE receiving TH in the Canton of Zurich, Switzerland, and was therefore limited by this small retrospective study population based on limited subject selection and low power confined to a high-income country with comparatively low heterogenicity of ethnic, economic, geographic and racial factors. Despite the low overall heterogenicity in this local setting, we failed to obtain reliable data on contributing factors of intersectionality and social, structural and environmental drivers of health in this vulnerable population of pregnant women and their neonates. This significantly limited the generalizability of both our results per se and the interpretation of these results. Moreover, multi-national studies that reflect high-income countries (including healthcare deserts) and low–middle-income countries would offer greater generalizability to be applied to healthcare choices.

### 4.5. Conclusions

This retrospective, multi-center cohort study provided valuable insights into the management of neonates with moderate-to-severe HIE receiving TH in the Canton of Zurich, Switzerland. The study revealed that while patient-related factors such as maternal, pregnancy and neonatal characteristics were similar across different levels of neonatal care, healthcare organizational factors—including response time, staffing, and transport logistics—played a significant role in management. The findings supported that the different delivery facilities were aware of their setting and opportunities and adapted their perinatal management according to their facility. Despite similar HIE incidence across levels of neonatal care, the study reinforced a multifaceted approach, including the importance of standardized care practices and ongoing quality improvement initiatives, particularly in training neonatal resuscitation teams and enhancing communication across medical teams. Future research should focus on refining early identification and intervention strategies and improving documentation practices. The findings emphasized that, while neonatal care levels provided a framework for resource allocation, the key to improving management of neonates with HIE lies in the integration of human factors, system preparedness and adherence to evidence-based protocols. Moreover, evidence-based adjunct neuroprotective measures in addition to TH and comprehensive multidisciplinary collaborations along the maternal–fetal triad are currently the most important research areas, hopefully leading to a better future for affected neonates with HIE.

## Figures and Tables

**Table 1:** Incidence of HIE receiving TH by level of neonatal care at place of delivery.

	Birthing Center	Level I Neonatal Care	Level IIB Neonatal Care	Level III Neonatal Care
Facilities (n)	2	14	4	1
Hypothermia patients (n)	5	79	80	9
Deliveries (n)	4011	82,647	67,321	24,502
Patients receiving therapeutic hypothermia				
per 1000 live births (n)	1.25	0.96	1.19	0.37
Average annual rate per facility (mean, range)	0.28 (0.22–0.33)	0.67 (0.11–1.50)	2.22 (0.22–3.11)	1.00 (1.00–1.00)

Notes: live births in delivery facilities between 2015 and 2023. Registered neonates with HIE receiving TH in one of three neonatal cooling centers in the Canton of Zurich, Switzerland.

**Table 2:** Maternal, delivery and neonatal characteristics.

Characteristics	Level I (n = 79)	Level IIB (n = 80)	
	n (%) or Median (IQR)	n (%) or Median (IQR)	*p* Value
**Infant characteristics**			
Sex, male	42 (53.2)	45 (56.3)	0.817
Gestational age (weeks)	39.6 (38.2, 40.8)	40.1 (39.0, 40.7)	0.459
Birth weight (grams)	3250 (2925, 3558)	3400 (3000, 3700)	0.303
**Maternal characteristics**			
Maternal age (years)	33 (29, 36)	33 (31, 35)	0.204
Multiple gestation (*twins in all cases*)	5 (6.3)	1 (1.3)	0.117
Primiparae	40 (51.9)	52 (65.0)	0.134
Adpositas	6 (7.6)	6 (7.5)	1.000
Maternal medical diagnosis	46 (58.2)	51 (63.8)	0.582
Maternal nicotine	4 (5.1)	5 (6.3)	1.000
Maternal alcohol	0 (0.0)	0 (0.0)	NA
Maternal cannabis/other drugs	0 (0.0)	0 (0.0)	NA
Mother from outside a German-speaking country *	21 (26.6)	20 (25.0)	0.858
..and without knowledge of a Swiss national language or English	5 (23.8)	7 (35.0)	0.506
Fertility treatment	5 (6.3)	4 (5.0)	0.746
Unregular pregnancy checkups	3 (3.8)	4 (5.0)	1.000
Maternal COVID-19 infection, peripartal	0 (0.0)	0 (0.0)	NA
Maternal polytrauma	0 (0.0)	0 (0.0)	NA
Oligohydramnion	0 (0.0)	9 (11.3)	0.003
Polyhydramnion	4 (5.1)	1 (1.3)	0.210
**Delivery characteristics**			
Delivery on weekend (Saturday/Sunday)	22 (27.8)	28 (35.0)	0.424
Delivery at night time (08:00 pm to 07:59 am)	45 (57.0)	45 (56.3)	1.000
Mode of delivery			
Vaginal delivery (all)	41 (51.9)	42 (53.2)	1.000
spontaneous, cephalic	23 (29.1)	20 (25.3)	
spontaneous, breech	1 (1.3)	1 (1.3)	
Instrumental	17 (21.5)	21 (26.6)	
Cesarean delivery (all)	38 (48.1)	37 (46.8)	1.000
elective CS	3 (3.8)	0 (0.0)	
emergency CS	19 (24.1)	24 (30.4)	
secondary CS	16 (20.3)	13 (16.5)	
Cardiotocography used	68 (98.6)	68 (98.6)	1.000
Pathological cardiotocogram	39 (72.2)	49 (75.4)	0.856
Delivery			
Known delivery complication ^#^	29 (45.3)	22 (31.0)	0.124
Delayed acces to medical-obstetric care	4 (5.1)	6 (7.5)	0.746
Unusual delivery setting	4 (5.1)	4 (5.0)	1.000
Refusal of recommended medical measures	4 (5.1)	4 (5.0)	1.000
Presence of medical specialists			
Obstetric physician in-house	79 (100.0)	80 (100.0)	1.000
Pediatric physician in-house	4 (5.1)	69 (86.3)	<0.001
Anesthesiologic staff present at delivery	64 (92.7)	57 (91.9)	1.000

Notes: CS, cesarean section; NA, not applicable. * German-speaking countries are Austria, Germany and Switzerland. ^#^ Delivery complication, i.e., perinatal sentinel event, was defined as at least one of the following: Head entrapment, placental abruption, prolapsed cord, ruptured uterus, and shoulder dystocia. Missing data: parity = 2 in the Level I group. Maternal age = 1 and mode of delivery = 1 in the Level IIB group. Cardiotocography = 10 and 11, pathological cardiotocogram = 15 and 4, delivery complication = 15 and 9, and anesthesiologic presence = 10 and 18, in the Level I and Level IIB group, respectively.

**Table 3:** Perinatal characteristics.

	Level I (n = 79)	Level IIB (n = 80)	
	n (%) or Median (IQR)	n (%) or Median (IQR)	*p* Value
Apgar score			
at 1 min	1 (0, 2)	2 (1, 3)	0.223
at 5 min	3 (1, 5)	4 (2, 5)	0.427
at 10 min	4 (2, 6)	5 (3, 7)	0.251
Umbilical artery pH	6.96 (6.80, 7.12)	7.01 (6.88, 7.13)	0.267
Resuscitation in the delivery room *	51 (64.6)	35 (44.9)	0.020
Blood gas analysis, worst ^#^			
pH	6.83 (6.76, 6.94)	6.87 (6.79, 6.96)	0.462
Base deficit (mmol/L)	19.0 (15.8, 23.2)	17.0 (14.5, 20.6)	0.109
Lactate (mmol/L)	13.2 (11.4, 16.0)	13.4 (11.2, 15.0)	0.401

Notes: * Resuscitation was not clearly defined before 2020. From 2020 on, resuscitation was defined as any form of ventilation necessary at the age of 10 min. ^#^ Worst blood gas analysis was defined as worst blood gas results within 60 min after birth, including umbilical gases. Missing data: Apgar at 1, 5 and 10 min = 1 and worst blood gas pH = 3 in the Level I group. Resuscitation = 2 in the Level IIB group. Umbilical artery pH = 15 and 3, worst blood gas base deficit = 5 and 5, and lactate = 10 and 3 in the Level I and Level IIB group, respectively.

**Table 4:** Variables on neonates with transport between birth facility and neonatal cooling center.

	Level I (n = 79)	Level IIB (n = 58)	
	Median (IQR)	Median (IQR)	*p* Value
Call of transport team (minute of life)	12 (7, 23)	34 (23, 50)	<0.001
Arrival of transport team (minute of life)	56 (44, 68)	74 (58, 93)	<0.001
Time from call to arrival (minutes)	40 (33, 48)	38 (28, 45)	0.084
Distance birth hospital to cooling center (kilometers)	22 (15, 31)	6 (6, 6)	<0.001
Age when cooling was initiated (hours)	3 (2, 4)	3 (2, 4)	0.431
Age when temperature ≤ 34 °C was reached (hours)	4 (3, 5)	4 (3, 5)	0.547

Notes: 22 neonates in Level IIB were excluded, as being born and treated with TH in-house. Age when cooling was initiated (hours) refers to starting the servo-controlled whole-body cooling device, with only passive cooling during neonatal transport to strictly avoid hyperthermia in the transport incubator. Missing data: age when cooling was initiated = 3 and age when target temperature was reached = 2, in the Level I group. Call time = 20 and 18, arrival time = 4 and 3, time from call to arrival = 21 and 18, in the Level I and Level IIB group, respectively.

**Table 5:** Neurologic prognostic factors.

Neurologic Variables	Level I (n = 79)	Level IIB (n = 80)	
	n (%) or Median (IQR)	n (%) or Median (IQR)	*p* Value
Sarnat Score			
before cooling			0.598
stage 1	5 (6.4)	3 (3.9)	
stage 2	51 (65.4)	47 (61.8)	
stage 3	22 (28.2)	26 (34.2)	
day 1			0.398
stage 1	16 (21.6)	22 (30.1)	
stage 2	37 (50.0)	36 (49.3)	
stage 3	21 (28.4)	15 (20.5)	
day 2 *			0.084
stage 1	16 (24.6)	27 (37.5)	
stage 2	36 (55.4)	39 (54.2)	
stage 3	13 (20.0)	6 (8.3)	
day 3 *			0.193
stage 1	19 (30.2)	31 (45.6)	
stage 2	37 (58.7)	31 (45.6)	
stage 3	7 (11.1)	6 (8.8)	
day 4 *			0.840
stage 1	24 (46.2)	32 (49.2)	
stage 2	25 (48.1)	28 (43.1)	
stage 3	3 (5.8)	5 (7.7)	
Seizure			
day 1	19 (24.1)	15 (18.8)	0.534
day 2 *	15 (19.7)	6 (7.7)	0.052
day 3 *	8 (11.1)	3 (3.9)	0.121
day 4 *	5 (7.2)	3 (3.9)	0.477
total	26 (32.9)	18 (22.5)	0.197
Highest lactate (mmol/L)			
day 1	8.2 (4.4, 13.6)	7.3 (4.3, 11.2)	0.533
day 2 *	2.7 (1.8, 4.7)	2.9 (2.1, 5.0)	0.313
day 3 *	1.9 (1.4, 3.1)	1.9 (1.3, 2.7)	0.571
day 4 *	1.4 (1.1, 2.1)	1.4 (1.2, 2.0)	0.937
Mortality	17 (21.5)	7 (8.8)	0.043

Notes: * The number of deaths on the first, second and third day of life was 1, 4 and 3 in the Level I group, and 1, 1 and 0 in the Level IIB group, respectively. This results in smaller cohort sizes on days 2, 3 and 4. Missing data: Sarnat before cooling = 1 and 4, Sarnat day 1 = 5 and 7, Sarnat day 2 = 13 and 7, Sarnat day 3 = 11 and 10, Sarnat day 4 = 19 and 13, seizures day 2 = 2 and 1, day 3 = 2 and 1, day 4 = 2 and 1, highest lactate on day 1 = 20 and 11, day 2 = 20 and 14, day 3 = 20 and 15, and day 4 = 20 and 14, in the Level I and Level IIB groups, respectively.

**Figure A1:**
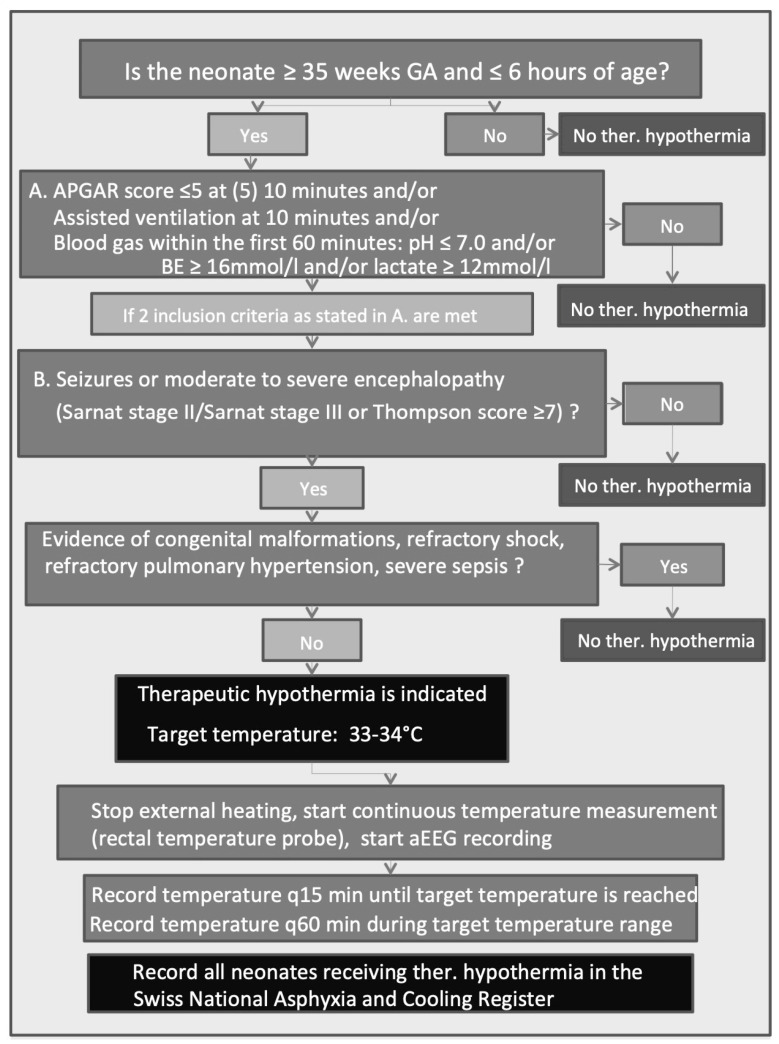
Flowchart for inclusion for TH.

## Data Availability

The datasets used and analyzed during the current study are available from the corresponding author on reasonable request.

## Supplementary Materials

The following supporting information can be downloaded at https://www.mdpi.com/article/10.3390/children13010142/s1, Table S1: Infant and perinatal characteristics, variables on initiation of therapeutic hypothermia and outcome of neonates born in birthing centers and the Level III neonatal care facility.

## Abbreviations

The following abbreviations are used in this manuscript:

HIE	Hypoxic–ischemic encephalopathy
TH	Therapeutic hypothermia
NICU	Neonatal intensive care unit
