# Supplementary material for: Differences in Management of Neonates with Hypoxic–Ischemic Encephalopathy (HIE) by Level of Neonatal Care Provided at Birth: Insights from a Referral-Based Cohort in the Canton of Zurich, Switzerland
Source: Children (Basel). 2026 Jan 19;13(1):142. doi: 10.3390/children13010142 (PMC12839900; doi:10.3390/children13010142)
Supplement: Supplementary file 1 [file children-13-00142-s001.zip › children-4054537-supplementary/Supplemental Table S1.pdf]

Supplemental Table S1: Infant and perinatal characteristics, variables on initiation of therapeutic hypothermia and outcome of neonates born in birthing centers and the Level III neonatal care facility.

	Birthing centers (n=5)	Level III (n=9)
	n (%) or median (IQR)	n (%) or median (IQR)
**Infant characteristics**		
Sex, male	3 (60.0)	5 (55.6)
Gestational age (weeks)	39.7 (39.3, 41.3)	38.6 (37.0, 39.7)
Birth weight (grams)	3400 (3300, 3740)	3000 (2680, 4000)
**Perinatal characteristics**		
Apgar score		
at 1 minute	2 (2, 3)	1 (0, 1)
at 5 minutes	4 (2, 6)	2 (1, 3)
at 10 minutes	5 (2, 5)	3 (2, 4)
Umbilical artery pH	N/A	6.93 (6.88, 7.18)
Resuscitation in the delivery room*	3 (60.0)	4 (44.4)
Blood gas analysis, worst#		
pH	7.01 (6.96, 7.09)	6.78 (6.75, 7.14)
Base deficit (mmol/L)	16.0 (15.4, 18.0)	16.8 (15.0, 20.5)
Lactate (mmol/L)	13.1 (10.8, 15.6)	15.0 (12.4, 17.0)
**Variables on therapeutic hypothermia**		
Age when cooling was initiated (hours)	3 (2, 5)	1 (1, 2)
Age when temperature ≤34°C was reached (hours)	3 (3, 5)	3 (3, 5)
Mortality	1 (20.0)	2 (22.2)

NA, not applicable.

* Resuscitation was not clearly defined before 2020. From 2020 on, resuscitation was defined as any form of ventilation necessary at the age of 10 minutes

# Worst blood gas analysis was defined as worst blood gas results within 60 min after birth, including umbilical gases.

Missing data: Worst blood gas pH = 2, worst lactate = 1 in the birthing center group. Worst blood gas base deficit = 2 and 1 in the birthing center and Level III group, respectively.
